# Epidemiology of Q Fever in Southeast Europe for a 20-Year Period (2002–2021)

**DOI:** 10.1007/s44197-024-00288-4

**Published:** 2024-09-04

**Authors:** Tatjana Pustahija, Snežana Medić, Vladimir Vuković, Zagorka Lozanov-Crvenković, Aleksandra Patić, Mirjana Štrbac, Verica Jovanović, Dragana Dimitrijević, Milunka Milinković, Mirjana Lana Kosanović, Helena C. Maltezou, Kassiani Mellou, Sanjin Musa, Marijan Bakić, Sanja Medenica, Nikolina Sokolovska, Nina Vukmir Rodić, Milica Devrnja, Mioljub Ristić, Vladimir Petrović

**Affiliations:** 1 Department of Epidemiology, Faculty of Medicine, University of Novi Sad, Novi Sad, Serbia; 2 Institute of Public Health of Vojvodina, Novi Sad, Serbia; 3 Department of Mathematics and Informatics, Faculty of Science, University of Novi Sad, Novi Sad, Serbia; 4 Department of Microbiology with Parasitology and Immunology, Faculty of Medicine, University of Novi Sad, Novi Sad, Serbia; 5 Institute of Public Health of Serbia, Belgrade, Serbia; 6 Andrija Stampar Teaching Institute of Public Health, Zagreb, Croatia; 7 Directorate for Research, Studies and Documentation, National Public Health Organization, Athens, Greece; 8 Department of Epidemiological Surveillance and Interventions, Hellenic Centre for Diseases Control and Prevention, Athens, Greece; 9 Department of Epidemiology, Institute for Public Health of the Federation of Bosnia and Herzegovina, Sarajevo, Bosnia and Herzegovina; 10 Sarajevo School of Science and Technology, Sarajevo, Bosnia and Herzegovina; 11 Institute of Public Health of Montenegro, Podgorica, Montenegro; 12 Epidemiology with Unit for Pest Control and Laboratory of Entomology, Center for Public Health, Skopje, North Macedonia; 13 Public Health Institute of the Republic of Srpska, Banja Luka, Bosnia and Herzegovina; 14 Faculty of Medicine, University of Banja Luka, Banja Luka, Bosnia and Herzegovina

**Keywords:** Q fever, *Coxiella burnetii*, Epidemiology, Southeast Europe, Surveillance

## Abstract

This study aimed to assess epidemiological trends of Q fever in six countries of Southeast Europe by analysing surveillance data for 2002–2021 period. In this descriptive analysis, we collected and analysed data on confirmed human Q fever cases, obtained from the national Public Health Institutes of Bosnia and Herzegovina, Croatia, Greece, Montenegro, North Macedonia and Serbia. Overall, 2714 Q fever cases were registered during the 20-year period. The crude average annual notification rate was 0.82 (± 2.06) (95% CI: 0.47–1.16) per 100,000 inhabitants, ranged from 0.06 (± 0.04) (95% CI: 0.04–0.08) /100,000 in Greece to 2.78 (± 4.80) (95% CI: 0.53–5.02) /100,000 in the Republic of Srpska (entity of Bosnia and Herzegovina). Significant declining trends of Q fever age standardized rates were registered in Croatia, the Federation of Bosnia and Herzegovina, North Macedonia and Serbia, with an average annual change of -30.15%; -17.13%; -28.33% and − 24.77%, respectively. An unequal spatial distribution was observed. The highest average age-specific notification rate was reported in the 20–59 age group (0.84 (± 0.40) (95% CI: 0.65–1.02) /100,000). Most cases (53.69%) were reported during the spring. Q fever remains a significant public health threat in this part of Europe. The findings of this study revealed the endemic maintenance of this disease in the including countries, with large regional and subnational disparities in notification rates. A downward trend was found in Q fever notification rates across the study countries with the average notification rate higher than in the EU/EEA, during the same period.

**Supplementary Information:**

The online version contains supplementary material available at 10.1007/s44197-024-00288-4.

## Introduction

Query (Q) fever is a worldwide distributed infection of zoonotic origin, caused by the intracellular Gram-negative bacterium *Coxiella burnetii (C. burnetii)* [1]. The World Health Organization (WHO) has identified *C. burnetii* as an agent of concern, classified by the Centres for Disease Control and Prevention (CDC) as a Category B biological agent [2, 3]. The large number of animal reservoirs, various routes of transmission, resistance of *C. burnetii* to environmental conditions, as well as the small infectious dose, contribute to the disease burden, especially in endemic areas [2, 4]. Infection in humans is usually asymptomatic, but mild to severe or rarely fatal disease may occur [1, 5]. Symptomatic human Q fever typically manifests as a flu-like illness, with potential complications including pneumonia or hepatitis. In a minority of cases (< 5%), progression to a chronic form of the disease may occur [4, 5]. Mammals, reptiles, birds and arthropods (mainly ticks) serve as a reservoir for Q fever [6, 7]. The main route of human infection is inhalation of contaminated aerosol or dust, originating from the placenta or body fluids of infected animals, primarily domestic ruminants (sheep, goats and cattle). Although interhuman transmission is very rare, transplacental, sexual and even nosocomial infections have been described [3, 7, 8].

Due to the epidemiological characteristics of Q fever, the geographical distribution of human Q fever varies. The disease can be endemic or hyperendemic in some areas, while large-scale outbreaks have occurred [9]. Q fever is an underestimated disease in many countries, due to an inadequate reporting system or misdiagnosis. As the WHO emphasizes, the large epidemics of Q fever in Europe indicate the potential risk that this disease will develop into a significant public health problem [10–12].

In Southeastern Europe (SEE) the first reports of Q fever (called “*Balkan Grippe*”) originates from the World War II [13, 14]. In this part of Europe, more precisely in the north of Serbia, in 1976, a large outbreak was registered with approximately 900 cases, at that time the largest in Europe [15]. In the past, the maintenance of Q fever in SEE was influenced by various factors, such as the unfavorable epizootiology situation in domestic ruminants, nomadic pastoralism and grazing system in sheep farming, and also the low effectiveness of general preventive measures and unawareness of livestock farmers [16]. The epidemiology of Q fever in this region of Europe is largely unknown. Hence, the aim of the study was to summarize the epidemiological characteristics of human Q fever in six SEE countries in the period 2002–2021, encompassing assessment of trends of the age standardized rates (ASR) of Q fever notifications across the participating countries as well as examination of factors potentially associated with Q fever in humans.

## Methods

### Study Setting

We conducted a chronological, demographic and topographical analysis of data, obtained from the national surveillance systems of six countries of SEE [Bosnia and Herzegovina (B&H) - two entities: the Federation of B&H and the Republic of Srpska, Croatia, Greece, Montenegro, North Macedonia and Serbia]. The study area spreads out over the territory of approximately 368 thousand square kilometers (km²), with an average population density of 73.44 inhabitants per km² in the observed period (range: 44.76 inhabitants/ km² in Montenegro- 82.58 inhabitants/ km² in Greece) [17–24].

### Characteristics of Q Fever Surveillance in SEE Countries

In accordance with the applicable Law, Q fever is a mandatory notifiable disease in all participating SEE countries.

In all involved countries, surveillance of Q fever is compulsory and comprehensive, on the national level. The surveillance is case-based and relies on the European Centre for Disease Prevention and Control (ECDC) case definition, encompassing a set of criteria used to classify cases (Supplementary Material Table S1) [25]. Accordingly, a confirmed case of Q fever has to meet at least one of the clinical criteria (fever, pneumonia and hepatitis) and at least one of the laboratory criteria (isolation of *C. burnetii* from a clinical specimen, detection of nucleic acid of *C burnetii* in a clinical specimen and determination of *C. burnetii* specific antibody response (IgM and/or IgG against phase II antigen).

For laboratory confirmation of the disease, serological tests were used [mainly enzyme-linked immunosorbent assay (ELISA), less often immunofluorescence assay (IFA)] (Supplementary Material Table S1). Although the polymerase chain reaction assay (PCR) is a fast and highly sensitive method, it was not available for routine diagnostics in the studied countries, during the observed period.

### Data Sources and Collection

Data on human Q fever cases were collected from the National Public Health Institutes (NPHI) databases of all SEE. The study covered a 20-year period, from January 1, 2002 to December 31, 2021, with the exception of Greece, where the data were available for the 18 year-period (2004–2021). Demographic data, which included census data and/or population estimates, were obtained from the websites of the National Statistical Institutes of each country and were used to calculate Q fever notification rates [17–24]. Age and sex data of the Q fever cases were not available for the Republic of Srpska in the period from 2002 to 2005, hence corresponding rates could not be calculated. Data on Q fever cases classified by administrative areas were provided from four countries: B&H (both entities), Croatia, Montenegro and Serbia. Data on hospitalization due to Q fever were collected only for Greece, Montenegro and the Republic of Srpska. The seasonal distribution of Q fever was analyzed for 2382 cases, based on the submitted number of reported cases per month. The data on the standard European population were extracted from the Eurostat database and The Scottish Health and Social Care open data platform [26–27].

### Data Analysis

Descriptive analyses were conducted to summarize the epidemiological characteristics of Q fever across all included SEE countries and years (2002–2021) (for Greece 2004–2021). The crude notification rates were calculated as the number of the Q fever cases divided by the number of people in the exposed population in the mid-year and expressed as the number of Q fever cases per 100,000 inhabitants. The average notification rates per 100,000 inhabitants were estimated with a corresponding standard deviation and confidence intervals (CIs). We performed the direct standardization according to the European population for calculation of ASR per 100,000 population. Trends of the ASR of Q fever notification rates across the study area and period were assessed using the joinpoint regression analysis that allowed the assessment of the measure and direction of the trend, using models fitted to data. We plotted the changes of ASR trends per each country and estimated the average annual percent change (AAPC) in the Q fever ASR with corresponding 95% CIs. We classified trend (increasing or decreasing) as statistically significant if the slope of the trend was statistically significant, i.e., if the AAPC is significantly different from zero at the alpha = 0.05 level. Due to the unavailability of data on the number of cases by age group for the period 2002–2005 in the Republic of Srpska, the trend was explored in the following period (2006–2021).

Besides, age-specific notification rates per 100,000 inhabitants were calculated for the age groups 0–19, 20–59 and ≥ 60 years. The male to female ratio (M/F) was examined across the entire study area and separately for all participating countries. The percentage of hospitalized patients for countries with available data, was also calculated.

Based on the month of notification, Q fever cases were divided into four meteorological seasons for the Northern Hemisphere: winter (December, January and February), spring (March, April and May), summer (June, July and August) and autumn (September, October and November) in accordance with the National Centers for Environmental Information (NCEI) definition [28]. Wilcoxon matched-pairs sign-rank test was used to test differences in age-specific notification rates, between age group by country and year of registration, and the corresponding average in age group of SEE, as well as to compare the average number of Q fever cases by meteorological season and the average number of cases during 2002–2021 by years. The seasonal distribution of Q fever by year for each country was further shown.

Distribution of Q fever cases according to the administrative area over the study period was presented in a map made using Quantum GIS software (QGIS), version 3.4.14. These data were mapped according to the average notification rate per 100,000 inhabitants, calculated based on the patients’ place of residence. The spatial distribution of Q fever cases in Croatia and Serbia was presented in relation to the statistical region of the third level (NUTS 3) consisting of 21 administrative units (20 counties and the City of Zagreb) and 30 administrative units (29 counties and the City of Belgrade), respectively. There are no available data about population and number of Q fever cases of Autonomous Province of Kosovo and Metohija, therefore it was not included in the study. Since Montenegro is divided into Local Administrative Units (LAU) according to the Nomenclature of Territorial Units for Statistics, we used LAU level 1 to display the spatial distribution of Q fever across this country. Bosnia and Herzegovina consists of two entities (the Federation of B&H and the Republic of Srpska) and the Brčko District. The Federation of B&H is further divided into 10 cantons, and this division is used for spatial presentation of Q fever in this entity. Whereas, to demonstrate the spread of Q fever on the territory of the Republic of Srpska, we relied on the division by six regions, which represent areas of jurisdiction of the IPH of the Republic of Srpska (Banja Luka-Bijeljina, Trebinje, Foča, Doboj, Zvornik and Istočno Sarajevo).

We examined factors potentially associated with the occurrence of Q fever cases, by comparison to the general population of SEE, using 2014 population estimate data in a bivariate analysis and calculating odds ratio (OR) with the corresponding 95% CIs. This analysis covered the period 2006–2021, due to the availability of all required data.

Statistical analyses were performed using Stata version 16 (StataCorp LLC. 2019) and the Joinpoint regression software version 5.02. The p-value < 0.05 was considered statistically significant across the analyses.

## Results

**Fig. 1:**
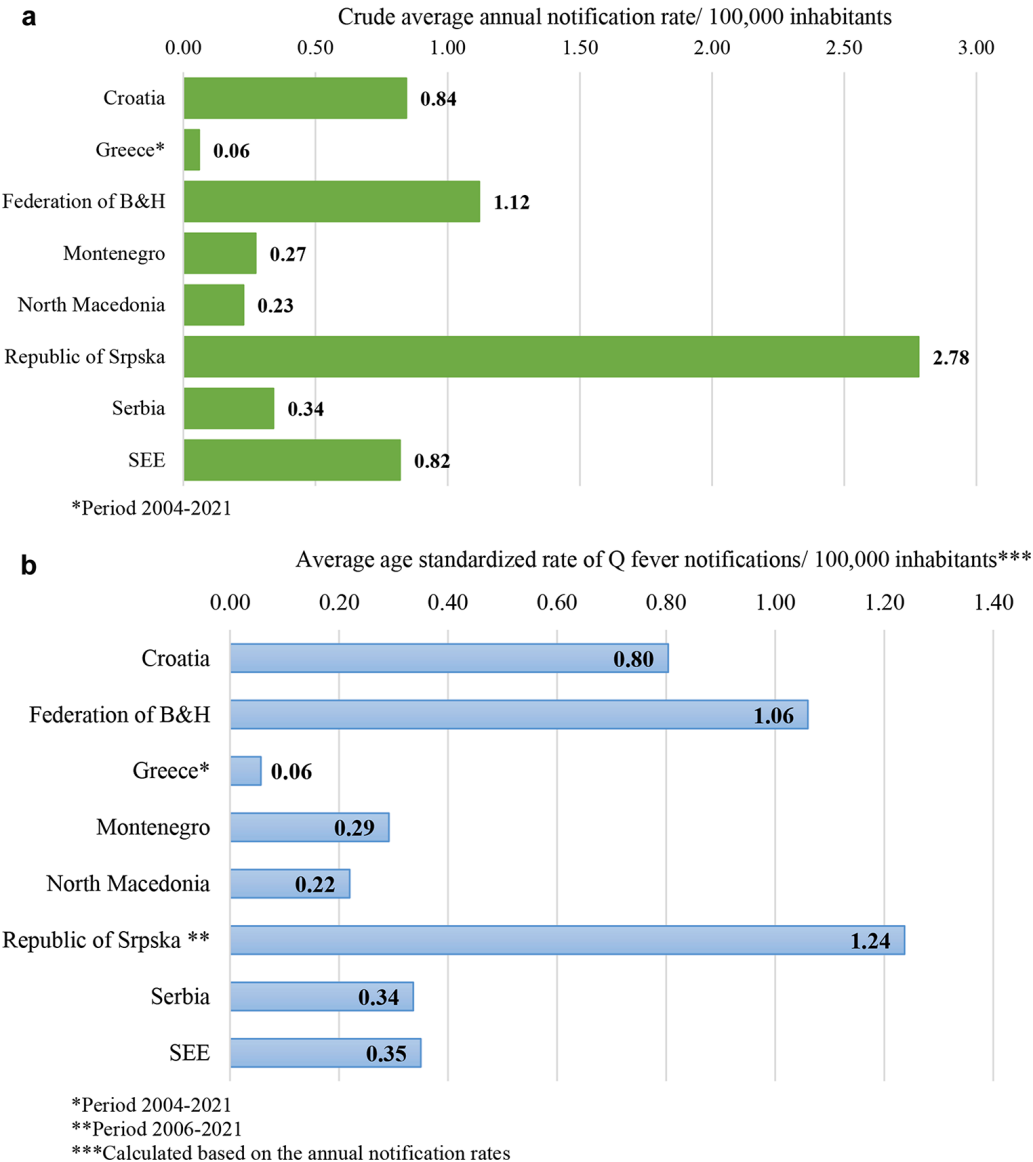
(**a**) The crude average annual notification rate of Q fever by SEE countries and B&H entities, 2002–2021. (**b**) Average age standardized notification rate of Q fever of SEE countries including B&H entities, 2002–2021

A total of 2714 human Q fever cases, including one death outcome, were reported in six countries of SEE during the 20-year study period [case fatality rate (CFR) = 0.04%]. One fatal case of Q fever was recorded in the Republic of Srpska in 2003 (CFR = 0.13%). The crude average annual notification rate of Q fever in the study area and period was 0.82 (± 2.06) (95% CI: 0.47–1.16) per 100,000 inhabitants (ranging from 0.05/100,00 in 2021 to 3.36/100,000 in 2004). The lowest crude average annual notification rate was 0.06 (± 0.04) (95% CI: 0.04–0.08)/100,000 in Greece and the highest: 2.78 (± 4.80) (95% CI: 0.53–5.02) /100,000 in the Republic of Srpska (Fig. 1a).

The average age standardized rates of Q fever notifications peaked in the B&H entities the Republic of Srpska and the Federation of B&H (1.24/100,000 and 1.06/100,000, respectively) (Fig. 1b).

In the countries that provided data, the percentage of hospitalized cases varied in the range from 19.32% in the Republic of Srpska to 91.23% in Greece (Table 1).

**Table 1:** Average annual percent change in the ASR of Q fever notifications (per 100,000) and other epidemiological indicators across the included SEE countries, 2002–2021

Country	AAPC (%)	95% LCI*	95% UCI*	Percentage of hospitalized cases (%)	M/F ratio**	Month with highest number of cases	Percentage of administrative areas reporting at least one case per year (%)	Period analysed
Croatia	-30.15*	-35.14	-24.97	ND	3.43: 1	February	86.36	2002–2021
Federation of B&H	-17.13*	-21.7	-13.3	ND	6.51: 1	May	90.00	2002–2021
Greece	12.33*	0,11	26.1	91.23	2.56: 1	October	ND	2004–2021
Montenegro	-3.55	-30.29	35	32.35	1.13: 1	June	59.09	2002–2021
North Macedonia	-28.33*	-36.73	-21.32	ND	2.71: 1	April, May, June	ND	2002–2021
Republic of Srpska	-3.56	-14.73	9.24	19.32	3.25: 1	May	85.71	2006–2021
Serbia	-24.77*	-32.63	-16.31	ND	2.36: 1	February, March	43.33	2002–2021
SEE	-14.20*	-18.15	-9.28	NA	3.35: 1	May	NA	2002–2021

*LCI- Lower 95% confidence interval

UCI- Upper 95% confidence interval

**Unavailable data for the Republic of Srpska in the period 2002–2005

ND- No data

NA- Not applicable

**Fig. 2:**
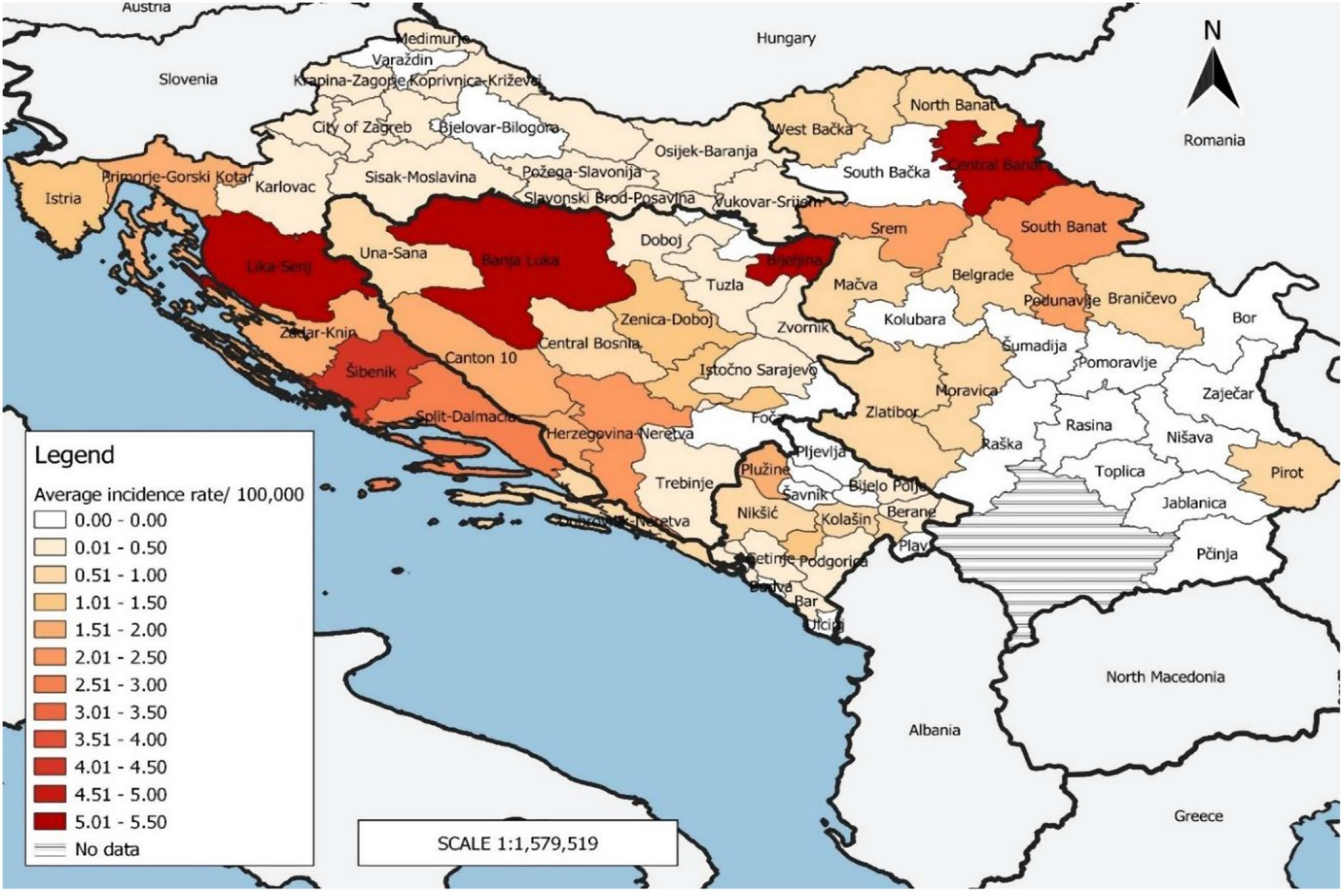
Distribution of Q fever cases by the administrative area in four SEE coutries (B&H, Croatia, Montenegro, Serbia), 2002–2021

An unequal spatial distribution of Q fever was noted across administrative levels of four SEE countries with available data. The highest average notification rate of 5.32 (± 5.18) (95% CI: 0.69–9.89) /100,000 was recorded in the cities of Banja Luka and Bijeljina in B&H (the Republic of Srpska), followed by Lika-Senj county in Croatia and Central Banat district of Serbia [5.28 (± 7.38) (95% CI: 1.82–8.73)/100,000; 5.04 (± 5.18) (95% CI: 2.37–7.70) /100,000; respectively]. Excluding areas where no Q fever cases are registered in the study period, in the another entity of B&H (the Federation of B&H), the notification rate ranged from 0.49 (± 0.91) (95% CI: 0.06–0.92)/100,000 in Tuzla canton to 2.31 (± 2.15) (95% CI: 1.30–3.31) /100,000 in Herzegovina-Neretva canton. In Montenegro, the highest notification rate was recorded in the municipality Plužine [1.86 (± 8.32) (95% CI: -2.03–5.75) /100,000] and the lowest was in the municipality Bar [0,11 (± 0.51) (95% CI: -0.13–0.36) /100,000] (Fig. 2). The percentage of administrative units that reported at least one case of Q fever per year was the highest in the Federation of B&H (90.00%), and the lowest in Serbia (43.33%) (Table 1).

According to the results of joinpoint regression analysis, ASR od Q fever notifications revealed statistically significant decreasing trend, across SEE (AARC=-14.20%) during the study period. Observing by countries, statistically significant downward trends in the period 2002–2021 were registered in Croatia, the Federation of B&H, North Macedonia and Serbia, with an average change in ASR of -30.15%; -17.13%; -28.33% and − 24.77% per year, respectively. In Greece, ASR reveled an upward trend in the period 2004–2021. The Q fever ASR trend was stable in Montenegro from 2002 to 2021 and in the B&H entity the Republic of Srpska in the period 2006–2021 (Table 1).

**Fig. 3:**
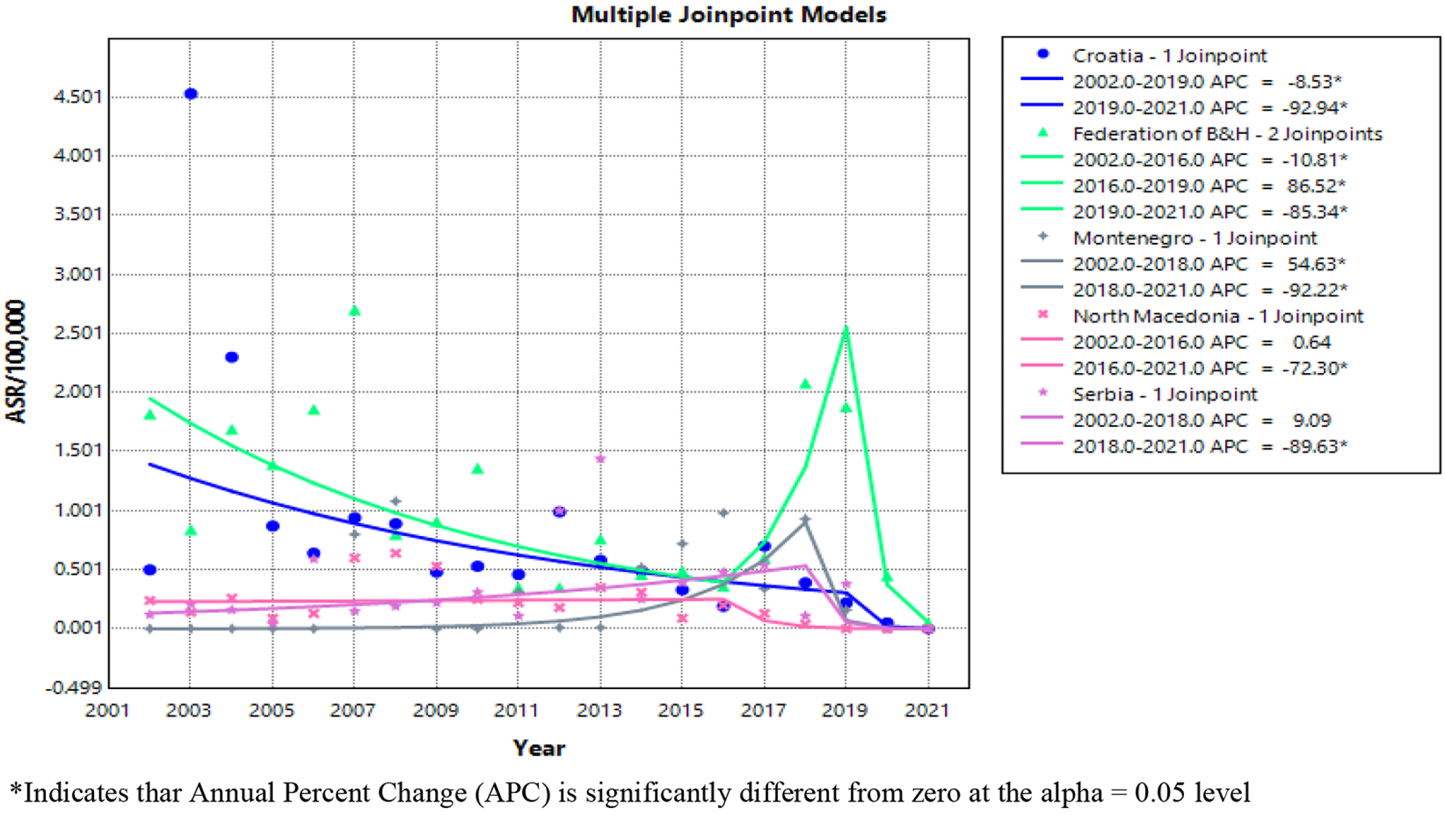
Joinpoint regression analysis plot of Q fever ASR trends for five SEE countries (Croatia, the Federation of B&H, Montenegro, North Macedonia and Serbia), 2002–2021

The results of the joinpoint regression analysis of the Q fever ASR for five countries of SEE, with available data for the period from 2002 to 2021, are further depicted in Fig. 3. In Croatia, the ASR trend decreased statistically significantly by 8.53% per year in the period 2002–2019, and then, in the following period, the ASR notably declined by 92.94% annually. In the Federation of B&H, a significant decline in the ASR of 10.81% annualy was observed in the period from 2002 to 2016, followed by an significant increase of 86.52% per year in the period 2016–2019 and subsequent annual decline of ASR by 85.34% in the period 2019–2021. In Montenegro, a remarkable annual rise in Q fever ASR of 54.63% was noticed in the period 2002–2018, but a significant decline of 92.22% per year occurred during the period 2018–2021. In North Macedonia a significant change in the ASR of -72.30% annualy was spotted during the period from 2016 to 2021, without significant alterations in the Q fever ASR trend before that period. In Serbia, the Q fever ASR trend was stable until 2019, but in the subsequent period (2019–2021), a significant decrease of 89.63% per year was registered.

**Fig. 4:**
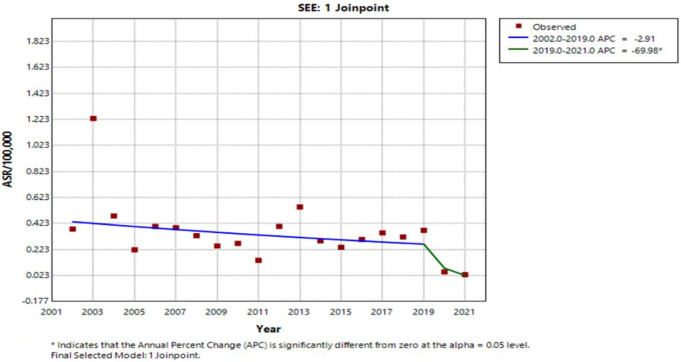
Joinpoint regression analysis plot of Q fever ASR trends for SEE countries, 2002–2021

Spotting the entire study area, the Q fever ASR trend was stable without substantial changes in the period until 2019. In the following period (2019–2021), statistically significant annual decline of 69.98% occurred (Fig. 4).

Overall male to female ratio in the six SEE countries was 3.35:1, and ranged from 1.13:1 in Montenegro to 6.51:1 in the Federation of B&H (Table 1).

In the entire study area, the highest average age-specific notification rate of 0.84 (± 0.40) (95% CI: 0.65–1.02) /100,000 was reported in the 20–59 age group (range: 0.03/100,000 in 2021–1.72/100,000 in 2003), while the lowest was reported in the age group of 0–19 years [0.18 (± 0.15) (95% CI: 0.11–0.25) /100,000]. Observed by country, the highest average age-specific notification rate in the age group 20–59 were recorded in Croatia [1.20 (± 1.44) (95% CI: 0.53–1.88) /100,000], the Federation of B&H [1.71 (± 1.25) (95% CI: 1.13–2.30) /100,000], North Macedonia [0.34 (± 0.28) (95% CI: 0.21–0.47) /100,000], the Republic of Srpska [1.74 (± 1.21) (95% CI: 1.10–2.39) /100,000] and Serbia [0.47 (± 0.44) (95% CI: 0.24–0.68) /100,000]. In Greece and Montenegro, the average age-specific notification rates of Q fever were highest in the age of 60 year and over [0.09 (± 0.08) (95% CI: 0.05–0.13) /100,000 and 0.39 (± 0.68) (95% CI: 0.07–0.70) /100,000)], respectively (Supplementary Material Table S2).

Wilcoxon matched-pairs signed-rank test revealed a statistically significant difference in the age group 20–59 years in Greece, Federation of B&H, Montenegro, North Macedonia, the Republic of Srpska and Serbia (*p* < 0.001; *p* = 0.002; *p* = 0.002; *p* < 0.001; *p* = 0.002; *p* = 0.005, respectively), when comparing the age-specific notification rate by country and year of registration with the corresponding average. The registered age-specific notification rates in the age group 20–59 years were significantly higher than the average in the Federation of B&H and the Republic of Srpska. A significant statistical difference in age-specific notification rates was also detected in the age group ≥ 60 years in Greece, North Macedonia, the Republic of Srpska and Serbia (*p* < 0.001; *p* < 0.001; *p* = 0.004; *p* < 0.001, respectively) and were lower than the average in all those countries, with the exception of the Republic of Srpska. The age-specific notification rates in age group 0–19 were statistically significantly lower than the average (*p* < 0.001; *p* = 0.024, respectively) in Greece and North Macedonia (Supplementary Material Table S2).

The majority of Q cases (53.69%), during 20-year study period, were registered in the spring. Compared to the average number of Q fever cases, registered during the entire period of the study, the average number of cases during the spring months was statistically significantly higher (*p* < 0.001), while the average number of cases during the summer and autumn was significantly lower (*p* = 0.0072; *p* < 0.001, respectively) (Table 2). Except Greece and Montenegro, in all included countries, the highest percentage of Q fever cases was recorded during the spring season (range: 36.59–75.59%). In Montenegro, the highest percentage of cases (47.06%) was reported in the summer, while in Greece, the most frequent notifications (29.82%) were in the autumn (Supplementary Material Table S3).

**Table 2:** Number of Q fever cases by season of case registration across the SEE countries, 2002–2021

	Winter *N* (%)	Spring (*N* (%)	Summer *N* (%)	Autumn *N* (%)	Average by year	Total *N* (%)
2002*	6 (2.44)	228 (92.68)	10 (4.07)	2 (0.81)	62	246 (100.00)
2003	122 (53.28)	93 (40.61)	6 (2.62)	8 (3.49)	57	229 (100.00)
2004	28 (6.98)	307 (76.56)	57 (14.21)	9 (2.24)	100	401 (100.00)
2005	17 (23.61)	45 (62.50)	6 (8.33)	4 (5.56)	18	72 (100.00)
2006	36 (35.29)	43 (42.16)	20 (19.61)	3 (2.94)	26	102 (100.00)
2007	17 (22.08)	42 (54.55)	14 (18.18)	4 (5.19)	19	77 (100.00)
2008	33 (31.73)	46 (44.23)	17 (16.35)	8 (7.69)	26	104 (100.00)
2009	9 (13.24)	29 (42.65)	22 (32.35)	8 (11.76)	17	68 (100.00)
2010	14 (28.00)	27 (54.00)	5 (10.00)	4 (8.00)	13	50 (100.00)
2011	33 (50.77)	21 (32.31)	10 (15.38)	1 (1.54)	16	65 (100.00)
2012	47 (35.34)	56 (42.11)	16 (12.03)	14 (10.53)	33	133 (100.00)
2013	31 (17.03)	48 (26.37)	83 (45.60)	20 (10.99)	46	182 (100.00)
2014	21 (22.58)	41 (44.09)	17 (18.28)	14 (15.05)	23	93 (100.00)
2015	22 (28.21)	32 (41.03)	10 (12.82)	14 (17.95)	20	78 (100.00)
2016	30 (28.30)	45 (42.45)	21 (19.81)	10 (9.43)	27	106 (100.00)
2017	12 (10.17)	69 (58.47)	18 (15.25)	19 (16.10)	30	118 (100.00)
2018	14 (13.21)	36 (33.96)	37 (34.91)	19 (17.92)	27	106 (100.00)
2019	18 (15.00)	58 (48.33)	23 (19.17)	21 (17.50)	30	120 (100.00)
2020	9 (39.13)	9 (39.13)	4 (17.39)	1 (4.35)	6	23 (100.00)
2021	2 (22.22)	4 (44.44)	2 (22.22)	1 (11.11)	2	9 (100.00)
Total	521 (21.87)	1279 (53.69)	398 (16.71)	184 (7.72)	NA	2382 (100.00)
Average by season	26	64	20	9	30	NA
p-value**	0,6541	**0**,**0001**	**0**,**0072**	**0**,**0001**	ref.	NA

* Winter 2002 includes number of Q fever cases from January to February 2002

**Wilcoxon matched-pairs sign-rank test comparing average number of cases by season and average number of cases during the period 2002–2021 by years

NA-Not applicable

2382 cases were analyzed

In bivariate analysis, it was found that during the period 2006–2021, residents of SEE countries, aged under 20 and over 59 years, compared to population belonging to the age group of 20–59 years, had a significantly lower probability of contracting Q fever. The probability decreased by 74.10% (OR = 0.259; *p* < 0.0001) for age group 0–19 and by 59.22% (OR = 0.4078; *p* < 0.0001) for age group ≥ 60 years. Besides, belonging to the female sex reduced the probability of contacting Q fever for 68.16% (OR = 0.3184; *p* < 0.0001). Compared to residents of Serbia, residents of the Republic of Srpska, Federation of B&H and Croatia had 3.39 (*p* < 0.0001), 2.57 (*p* < 0.0001) and 1.28 (*p* < 0.0005) times higher probability of contracting Q fever, while the probability for residents of Greece and North Macedonia was statistically significanly lower by 84.3% (OR = 0.1574; *p* < 0.001) and 39.7% (OR = 0.603; *p* < 0.001) (Table 3).

**Table 3:** Determinants of Q fever in confirmed cases compared to the general population in SEE countries, 2006–2021

	Study population (*N*; %)	General population (*N*; %)*	OR	95% CI	*p*-value
**Age**					
0–19	123 (7.54)	5,795,442 (20.43)	0.259	0.2153–0.3117	**< 0.0001**
20–59	1273 (78.05)	15,537,842 (54.77)	ref.	ref.	ref.
≥ 60	235 (14.41)	7,034,166 (24.80)	0.4078	0.3548–0.4687	**< 0.0001**
** Total**	**1631 (100.00)**	**28**,**367**,**450 (100.00)**	-	-	**-**
**Sex**					
Male	1222 (74.92)	13,830,435 (48.75)	ref.	ref.	ref.
Female	409 (25.08)	14,537,015 (51.25)	0.3184	0.2847–0.3562	**< 0.0001**
** Total**	**1631 (100.00)**	**28**,**367**,**450 (100.00)**	-	-	**-**
**Country of residence**					
Croatia	345 (21.15)	4,238,044 (14.94)	1.2844	1.116–1.4776	**0.0005**
Greece	109 (6.68)	10,926,698 (38.52)	0.1574	0.1277–0.194	**< 0.0001**
Federation of B&H	361 (22.13)	2,215,997 (7.81)	2.5702	2.2381–2.9516	**< 0.0001**
Montenegro	34 (2.08)	621,487 (2.19)	0.2421	0.1708–0.343	0.4061
North Macedonia	79 (4.84)	2,067,058 (7.29)	0.603	0.4748–0.758	**< 0.0001**
Republic of Srpska	251 (15.39)	1,166,831 (4.11)	3.3939	2.9086–3.9601	**< 0.0001**
Serbia	452 (27.71)	7,131,335 (25.14)	ref.	ref.	ref.
** Total**	**1631 (100.00)**	**28**,**367**,**450 (100.00)**	-	-	-

*Source: population estimate data 2014

## Discussion

Given the burden of emerging zoonoses, the importance of high-quality surveillance, cross-border cooperation and information exchange among member states was recognized and highlighted by the WHO [29]. Q fever is a neglected disease of great public health importance. To the best of our knowledge, this is the first study summarizing the epidemiological characteristics of Q fever during two decades in six countries of the southeastern part of Europe. The disease is compulsory notifiable in all participating countries, and showed large-scale regional and subnational disparities. Q fever ASR decline of about 14% per year during the observed period. In addition, Q fever was more common in males and the working age population.

During the 20-year period covered by this study, the registered crude average annual notification rate of 0.82 per 100,000 inhabitants in six countries of SEE was almost three times higher compared to the average notification rates reported in the European Union and the countries of the European Economic Area (EU/EEA) in the same period [30–34]. The average annual notification rates notified in Montenegro, North Macedonia and Serbia were at the level of the European average, while the Q fever notification rate in the Republic of Srpska and the Federation of B&H, was ten and four times higher than in EU/EEA, respectively. In contrast to the other countries surveyed, Greece recorded notification rates almost five times lower than those reported in the EU/EEA during the same period.

According to the ECDC data, the average Q fever ASR amounted to 0.28/100,000 in the EU/EEA in the period from 2008 to 2019. In the same period, an extremely high average ASR of 1.8/100,000 inhabitants was recorded in the Netherlands and was the result of the exceptionally large outbreak with over 4,000 cases recorded between 2007 and 2010 [35]. In the post-Q fever-epidemic period (2011–2022), this country reports low ASRs, ranging from 0.03 to 0.48 [30]. Except for the Netherlands, the highest average ASR of Q fever notification in the countries of EU/EEA were reported by Spain, Bulgaria and Hungary (0.60/100,000; 0.45/100,000; 0.43/100,000, respectively) [30].

The high percentage of hospitalized Q fever cases in Greece, Montenegro and the Republic of Srpska indicates the deficiency of passive surveillance and testing in the community and more frequent recognition of severe forms of the disease, that require hospital treatment [36]. Interestingly, the percentage of hospitalized patients with Q fever in these three countries was inversely proportional to the notification rate. This could indicate a lack of awareness about this disease at the primary level of health care in countries with a low Q fever notification rate [37]. Similar to our results, a high percentage of hospitalization was found in the United States of America (USA) during the period 2000–2012 and in the Northern Serbian province of Vojvodina between 2006 and 2015 [15, 36]. The percentage of hospitalized patients in the Netherlands outbreak ranged from 20 to 50% [38].

Large-scale variations in the spatial distribution of Q fever were observed in four SEE countries. In some parts of these countries, during the 20-year period, not a single case of the disease was reported, while in some areas of B&H, Croatia and Serbia, the average notification rate reached values of over 5 cases per 100,000 inhabitants. The mentioned differences in the spatial distribution of Q fever in SEE can be explained by the epidemiological characteristics of this disease, conditioned by the presence of the reservoir of *C. burnetii* and favourable environmental factors [39]. Besides, differences in the quality of surveillance of infectious diseases, i.e., existence of variations in the recognition of this disease by local physicians due to the wide range of clinical presentations and Q fever unawareness, lab capacity issues and, finally, differences in underreporting rates contributed to this geographical distribution [40–42]. An additional explanation could be the diversity of *C. burnetii* strains [39]. Namely, in regions with circulation of *C. burnetii* strains that are more virulent, the participation of more severe infections is higher, and they are more often recognized in the health system. Substantial disparities in the geographic distribution of Q fever have also been found in many other published studies [39, 43–45].

According to our results, a declining trend characterized ASR of Q fever during the study period in SEE. By country, the highest annual trend declines of over 25% was found in Croatia and North Macedonia. Downward trends in Q fever ASR were spotted, as well, in the Federation of B&H and Serbia, while in Montenegro, ASR revealed stability in the observed period. The trend in the period 2006–2021 in the Republic of Srpska did not show statistically significant changes. This should be interpreted cautiously, since the analysis did not include the first years of the 20-year period in the Republic of Srpska, when high crude annual notification rates were recorded. Thus, it is unlikely that the ASR trend was stable in the entire observed period (2002–2021). Greece is the only SEE country where the Q fever ASR trend increased significantly during the observed period, that may be related to increased awareness of physicians.

After the end of the outbreak in the Netherlands, there was a marked decline in Q fever ASR in the EU/EEA countries, in the period 2010–2012. In the following years, until 2019, the trend remained relatively stable with a very slight increase and then significantly decreased in the COVID-19 pandemic period [30–34]. A substantial downtrend of over 85% annually in the period from 2019 to 2021 was also recorded in Croatia and the Federation of B&H and in the period from 2018 to 2021 in Montenegro and Serbia. This decline is potentially associated with underdiagnosing and consequent underreporting due to the unavailability of health care during the pandemic period, as resources were focused on COVID-19 [30, 31, 34, 46]. One of the possible explanations for the downward trend in the pre-pandemic period in the studied SEE countries could be the decrease in the number of livestock, which agrees with the study by Lai and colleagues [47]. In Australia, the decline in the notification rate of Q fever was influenced by government-funded National Q fever Management Program (NQFMP) from 2001 to 2006, which included screening and vaccination of at-risk human population. Reduced Q fever incidence by up to 50% was maintained until 2009, but it started to rise again [48]. As the reason for the renewed increase, the change in the epidemiology of Q fever in Australia is cited in the literature, arose as a consequence of the more frequent infection of farmers, among whom vaccination coverage was lower compared to abattoir workers [43, 44, 48]. Otherwise, the vaccine against Q fever is licensed only in Australia, while it is not used in other parts of the world due to possible post-vaccination hypersensitivity reactions [49]. Additionally, this incidence trend in Australia is explained by the heightened awareness of physicians and the introduction of enhanced surveillance in 2012 [43, 44, 48].

Sex-related differences were observed among human Q fever cases recorded in the study area, during the 20-year period. Namely, males were affected from three to six and a half times more often, compared to females, across SEE countries. The exception is Montenegro, where Q fever was registered almost equally in males and females, probably due to the fact that females in this country are largely involved in livestock and household care. The predominance of males in contracting Q fever resembling this, has been reported in the EU/EEA and documented by the results of other authors [15, 30, 39, 45, 50]. These differences could arise from the more frequent professional exposure of males and biological dissimilarities [39]. In this regard, it has been proven that the hormone 17β-estradiol limits the infection caused by *C. burnetii* and therefore has a protective effect on the development of a more severe clinical form of Q fever in females [51]. Furthermore, in males, the overexpression of the circadian Per2 gene occurs during the acute infection, which can also have an impact on the severe clinical status in those patients [45, 51, 52]. In Australia, the proportion of females affected increased over time in the period 1991–2014 and was driven mainly by the decline in Q fever cases among males, due to the success of the NQFMP, which mainly referred to occupations predominantly performed by males [44].

Observing the entire investigated region and study period, our results revealed that most Q fever patients were middle aged adults. These findings are consistent with other publish data [39, 42–44, 50, 53] and are explained by the fact that this group is more often in contact with reservoirs of infection, and they are more frequently exposed to the environmental risk factors [54]. The age distribution of Q fever had a similar pattern with a peak in the middle age in all SEE countries, except Greece and Montenegro, where the notification rate of the disease increases with age, similar to USA and England and Wales with the highest notification rates observed in the oldest age group [36, 45]. The predominance of older adults among Q fever cases in Greece and Montenegro, may be possibly caused by the low notification rate of this disease on the territories of those countries, as a consequence of misdiagnosis due to non-specific symptoms or due to lack of awareness, but also due to aging of the population engaged in animal breeding [37, 55]. Furthermore, older people more often have associated chronic diseases, which favour developing a more severe form of Q fever, which is more easily recognized by the health-care system [39, 56].

Specific seasonal pattern of Q fever was observed in most of the SEE countries, with the highest number of cases during the spring (March-May). A similar seasonal feature of Q fever has been observed in the USA and Spain, corresponding to the breeding season in livestock [36, 57]. In EU/EEA countries, the highest number of cases was reported during the spring/summer season [34]. The shift of the disease season from the winter months to the spring and summer is a consequence of the cessation of nomadic animal husbandry, in which winter lambing and shearing were practiced [37]. Performing these activities in the warmer and drier season further carries an increased risk of aerosolization and windborne spreading of rickettsia, which has been described by numerous published studies [15, 41, 58, 59]. Additionally, an increase in the number of Q fever cases in the spring can be explained by religious holidays and the practice of slaughtering often uncontrolled livestock in home conditions, without adhering to hygienic measures [60]. Deviation from the seasonal pattern of Q fever in Greece is unclear. Perhaps the reason should be sought in meteorological factors and differences in the occurrence of wind seasons.

The results of our study revealed that females, compared to males, and population aged under 20 and over 59 years, compared to persons belonging to the age group of 20–59, had a significantly lower probability for acquisition of Q fever in the period 2006–2021 across all SEE countries. Specific sex and age distribution associated with Q fever was also confirmed by bivariate analysis conducted in the study of *Thill* and colleagues in French Guiana, where being a male and between 30 and 59 years were identified as factors related to *C. burnetii* infection [59]. This is explained by the previously discussed hormonal and genetic differences, as well as inequalities in the exposure among these population groups [45, 52, 54]. In the study in French Guiana, the country with the highest notification rate of Q fever in the world, place of residence was distinguished as a risk factor for Q fever [59]. Our analysis showed that in relation to persons with a place of residence in Serbia, the inhabitants of Greece and North Macedonia had a lower probability for acquiring of this disease, while the probability for the residents of Croatia, the Federation of B&H and the Republic of Srpska was significantly higher. Interpretation of this result requires caution, due to differences in quality of reporting between countries. During the largest outbreak ever recorded in the Netherlands, residing within 1 km of the farm, where the infected animals were kept, amplified the risk of contracting Q fever by up to 46 times [61]. Apart from this, in published studies occupational and environmental exposure are cited as pivotal risk factors for infection with *C. burnetii* [55, 61, 62].

### Limitations of the Study

The results of our research were affected by incomplete surveillance data, which are the result of a long study period and the consequent lack of the data. In the participating countries, the infectious disease notification forms were archived differently. In some including countries, the forms are kept for up to 10 years, so access to the data from earlier years of the observed period was disabled or difficult. Since the forms are the most authoritative source of epidemiological data, occupational and environmental exposure patterns and data of Q fever outbreaks, were not collected. All of this data would be important for a more comprehensive assessment of the epidemiological situation of this disease in the observed region. Another limitation of the study is underreporting, arose as a consequence of misdiagnosis, unavailability of laboratory diagnostics, difficult differential diagnosis, especially in the period of the COVID-19 pandemic. Although Q fever is a mandatory notifiable disease in all participating countries, the disinterest and lack of awareness of physicians significantly affect the quality of reporting. Due to all the aforementioned limitations, but especially due to the lack of data from animal surveillance or genomic data, the results of these surveillance-based analyses should be interpreted very cautiously.

## Conclusion

The findings of this study revealed the endemic maintenance of Q fever in SEE, with large variations in the spatial distribution observed, in and between the countries. The overall registered average notification rate in the six SEE countries was almost three times higher than that recorded in the EU/EEA countries during the same period, mainly due to the high notification rates in B&H entities and Croatia. We found downward trend in notification rates of Q fever, with the largest annual decline recorded in Croatia and North Macedonia. In addition, Q fever was more common in men and the working-age population. In most including countries, the highest number of cases of Q fever recorded during spring. Q fever continues to pose a threat to public health in SEE countries. The results of this study can serve as a cornerstone for a detailed analysis of the environmental and other factors that contribute to the maintenance of human Q fever, including analysis of animal/genomic surveillance data in individual countries.

## Electronic Supplementary Material

Below is the link to the electronic supplementary material.

Supplementary Material 1

Supplementary Material 2

Supplementary Material 3

## Data Availability

No datasets were generated or analysed during the current study.
