# Supplementary material for: Epidemiology of Q Fever in Southeast Europe for a 20-Year Period (2002–2021)
Source: J Epidemiol Glob Health. 2024 Sep 4;14(3):1305–18. doi: 10.1007/s44197-024-00288-4 (PMC11442714; doi:10.1007/s44197-024-00288-4)
Supplement: Supplementary file 1 — Supplementary Material 1 [file 44197_2024_288_MOESM1_ESM.docx]

**Table S1** Characteristics of the surveillance system of Q fever in the included SEE countries

|  |  | |  | |  | |  | |  | |  | |  |  |
| --- | --- | --- | --- | --- | --- | --- | --- | --- | --- | --- | --- | --- | --- | --- |
| Country | | National coverage | | Active/ Passive | | Aggregated | | Case based | | Case definition | | Laboratory tests | | Years included |
| B&H (Federation of B&H) | | Yes | | Passive | | No | | Yes | | ECDC | | ELISA | | 2002-2021 |
| B&H (Republic of Srpska) | | Yes | | Passive | | No | | Yes | | ECDC | | ELISA | | 2002-2021 |
| Croatia | | Yes | | Passive | | No | | Yes | | ECDC | | ELISA/ IFA | | 2002-2021 |
| Greece | | Yes | | Passive | | No | | Yes | | ECDC | | IFA | | 2004-2021 |
| Montenegro | | Yes | | Passive | | No | | Yes | | ECDC | | ELISA | | 2002-2021 |
| North Macedonia | | Yes | | Passive | | No | | Yes | | ECDC | | ELISA | | 2002-2021 |
| Serbia | | Yes | | Passive | | No | | Yes | | ECDC | | ELISA/ IFA | | 2002-2021 |
