# Supplementary material for: Epidemiology of Q Fever in Southeast Europe for a 20-Year Period (2002–2021)
Source: J Epidemiol Glob Health. 2024 Sep 4;14(3):1305–18. doi: 10.1007/s44197-024-00288-4 (PMC11442714; doi:10.1007/s44197-024-00288-4)
Supplement: Supplementary file 2 — Supplementary Material 2 [file 44197_2024_288_MOESM2_ESM.docx]

**Table S2** Age-specific incidence rate of Q fever by the year of registration and SEE countries, 2002-2021

| **Country** | **Age group** | **2002** | **2003** | **2004** | **2005** | **2006** | **2007** | **2008** | **2009** | **2010** | **2011** | **2012** | **2013** | **2014** | **2015** | **2016** | **2017** | **2018** | **2019** | **2020** | **2021** | **p-value*** | **Average** |
| --- | --- | --- | --- | --- | --- | --- | --- | --- | --- | --- | --- | --- | --- | --- | --- | --- | --- | --- | --- | --- | --- | --- | --- |
| **Croatia** | **0-19** | 0.13 | 2.95 | 0.50 | 0.31 | 0.21 | 0.00 | 0.11 | 0.00 | 0.32 | 0.33 | 0.23 | 0.34 | 0.12 | 0.00 | 0.00 | 0.24 | 0.00 | 0.00 | 0.00 | 0.00 | 0.809 | 0.29 |
|  | **20-59** | 0.81 | 6.42 | 3.50 | 1.41 | 0.72 | 1.52 | 1.45 | 0.77 | 0.73 | 0.64 | 1.50 | 0.78 | 0.70 | 0.62 | 0.36 | 1.14 | 0.60 | 0.33 | 0.09 | 0.00 | 0.411 | 1.20 |
|  | **≥ 60** | 0.17 | 1.94 | 1.33 | 0.20 | 0.82 | 0.51 | 0.40 | 0.30 | 0.29 | 0.19 | 0.57 | 0.38 | 0.37 | 0.00 | 0.00 | 0.18 | 0.27 | 0.18 | 0.00 | 0.00 | 0.737 | 0.40 |
| **Greece** | **0-19** | ND | ND | 0.00 | 0.00 | 0.00 | 0.00 | 0.04 | 0.00 | 0.00 | 0.00 | 0.00 | 0.14 | 0.05 | 0.05 | 0.05 | 0.00 | 0.05 | 0.05 | 0.00 | 0.00 | **<0.001** | 0.02 |
|  | **20-59** | ND | ND | 0.03 | 0.00 | 0.03 | 0.00 | 0.03 | 0.05 | 0.00 | 0.05 | 0.08 | 0.08 | 0.12 | 0.12 | 0.05 | 0.04 | 0.12 | 0.11 | 0.04 | 0.02 | **<0.001** | 0.05 |
|  | **≥ 60** | ND | ND | 0.04 | 0.04 | 0.04 | 0.00 | 0.00 | 0.00 | 0.04 | 0.04 | 0.21 | 0.11 | 0.24 | 0.10 | 0.17 | 0.07 | 0.17 | 0.23 | 0.03 | 0.10 | **<0.001** | 0.09 |
| **Federation of B&H** | **0-19** | 0.00 | 0.00 | 0.50 | 0.17 | 0.35 | 0.35 | 0.18 | 0.00 | 1.30 | 0.00 | 0.19 | 0.00 | 0.00 | 0.20 | 0.00 | 0.00 | 0.44 | 0.45 | 0.00 | 0.00 | 0.717 | 0.21 |
|  | **20-59** | 3.44 | 1.59 | 2.98 | 2.02 | 3.10 | 4.55 | 1.31 | 1.46 | 1.93 | 0.54 | 0.46 | 1.32 | 0.86 | 0.63 | 0.47 | 1.02 | 3.24 | 2.79 | 0.56 | 0.00 | **0.002** | 1.71 |
|  | **≥ 60** | 0.00 | 0.00 | 0.00 | 1.12 | 0.56 | 0.82 | 0.27 | 0.53 | 0.26 | 0.25 | 0.25 | 0.24 | 0.00 | 0.45 | 0.44 | 0.21 | 1.04 | 1.22 | 0.59 | 0.19 | 0.478 | 0.42 |
| **Montenegro** | **0-19** | 0.00 | 0.00 | 0.00 | 0.00 | 0.00 | 0.00 | 0.60 | 0.00 | 0.00 | 0.00 | 0.00 | 0.00 | 0.00 | 0.00 | 0.00 | 0.65 | 0.65 | 0.00 | 0.00 | 0.00 | 0.064 | 0.09 |
|  | **20-59** | 0.00 | 0.00 | 0.00 | 0.00 | 0.00 | 0.59 | 0.88 | 0.00 | 0.00 | 0.58 | 0.00 | 0.00 | 0.58 | 0.59 | 1.47 | 0.00 | 1.49 | 0.30 | 0.00 | 0.00 | **0.002** | 0.32 |
|  | **≥ 60** | 0.00 | 0.00 | 0.00 | 0.00 | 0.00 | 1.92 | 1.89 | 0.00 | 0.00 | 0.00 | 0.00 | 0.00 | 0.83 | 1.61 | 0.79 | 0.77 | 0.00 | 0.00 | 0.00 | 0.00 | 0.627 | 0.39 |
| **North Macedonia** | **0-19** | 0.00 | 0.00 | 0.35 | 0.00 | 0.18 | 0.18 | 0.00 | 0.38 | 0.19 | 0.00 | 0.00 | 0.00 | 0.21 | 0.00 | 0.00 | 0.00 | 0.00 | 0.00 | 0.00 | 0.00 | **0.024** | 0.07 |
|  | **20-59** | 0.44 | 0.26 | 0.35 | 0.17 | 0.17 | 0.76 | 0.92 | 0.84 | 0.25 | 0.42 | 0.33 | 0.66 | 0.50 | 0.17 | 0.25 | 0.25 | 0.08 | 0.00 | 0.00 | 0.00 | **<0.001** | 0.34 |
|  | **≥ 60** | 0.00 | 0.00 | 0.00 | 0.00 | 0.00 | 0.62 | 0.59 | 0.00 | 0.29 | 0.00 | 0.00 | 0.00 | 0.00 | 0.00 | 0.25 | 0.00 | 0.00 | 0.00 | 0.00 | 0.00 | **<0.001** | 0.09 |
| **Republic of Srpska** | **0-19** | ND | ND | ND | ND | 0.47 | 0.00 | 0.71 | 0.24 | 0.47 | 0.00 | 0.00 | 0.00 | 0.86 | 0.00 | 0.00 | 0.92 | 0.00 | 0.95 | 0.00 | 0.49 | 0.140 | 0.32 |
|  | **20-59** | ND | ND | ND | ND | 2.29 | 1.52 | 2.16 | 1.78 | 1.27 | 0.00 | 0.25 | 1.85 | 2.80 | 1.26 | 4.28 | 3.20 | 2.10 | 2.77 | 0.17 | 0.17 | **0.002** | 1.74 |
|  | **≥ 60** | ND | ND | ND | ND | 0.73 | 0.36 | 1.09 | 0.73 | 0.00 | 1.09 | 0.00 | 2.09 | 0.68 | 0.67 | 1.97 | 1.94 | 0.95 | 2.82 | 0.00 | 0.31 | **0.004** | 0.96 |
| **Serbia** | **0-19** | 0.21 | 0.07 | 0.12 | 0.00 | 0.06 | 0.00 | 0.06 | 0.06 | 0.00 | 0.07 | 0.49 | 2.61 | 0.14 | 0.22 | 0.07 | 0.00 | 0.00 | 0.07 | 0.00 | 0.00 | 0.212 | 0.21 |
|  | **20-59** | 0.15 | 0.31 | 0.17 | 0.07 | 1.03 | 0.29 | 0.34 | 0.37 | 0.57 | 0.15 | 1.56 | 1.43 | 0.31 | 0.52 | 0.79 | 0.80 | 0.16 | 0.46 | 0.00 | 0.00 | **0.005** | 0.47 |
|  | **≥ 60** | 0.00 | 0.12 | 0.18 | 0.00 | 0.12 | 0.00 | 0.00 | 0.06 | 0.06 | 0.06 | 0.28 | 0.49 | 0.21 | 0.26 | 0.16 | 0.47 | 0.10 | 0.46 | 0.00 | 0.00 | **<0.001** | 0.15 |
| **Average** | **0-19** | 0.07 | 0.60 | 0.24 | 0.08 | 0.18 | 0.08 | 0.24 | 0.10 | 0.33 | 0.06 | 0.13 | 0.44 | 0.20 | 0.07 | 0.02 | 0.26 | 0.16 | 0.22 | 0.00 | 0.07 | ref | 0.18 |
|  | **20-59** | 0.97 | 1.72 | 1.17 | 0.61 | 1.05 | 1.32 | 1.01 | 0.75 | 0.68 | 0.34 | 0.60 | 0.88 | 0.84 | 0.56 | 1.10 | 0.92 | 1.11 | 0.97 | 0.12 | 0.03 | ref | 0.84 |
|  | **≥ 60** | 0.03 | 0.41 | 0.26 | 0.23 | 0.32 | 0.60 | 0.61 | 0.23 | 0.13 | 0.23 | 0.19 | 0.47 | 0.33 | 0.44 | 0.54 | 0.52 | 0.36 | 0.70 | 0.09 | 0.09 | ref | 0.34 |

*Wilcoxson matched-pairs sign-rank test between the specific incidence rate in the age groups by country and year of registration vs. the corresponding average in the age group of SEE

NA-Not applicable

ND-No data

In bold = statistically significant results at p<0.05
