# Supplementary material for: Epidemiology of Q Fever in Southeast Europe for a 20-Year Period (2002–2021)
Source: J Epidemiol Glob Health. 2024 Sep 4;14(3):1305–18. doi: 10.1007/s44197-024-00288-4 (PMC11442714; doi:10.1007/s44197-024-00288-4)
Supplement: Supplementary file 3 — Supplementary Material 3 [file 44197_2024_288_MOESM3_ESM.docx]

**Table S3** Number of Q fever cases by meteorological season of registration in SEE countries, 2002-2021

|  | **Winter** | **Spring** | **Summer** | **Autumn** |
| --- | --- | --- | --- | --- |
|  | **N (%)** | **(N (%)** | **N (%)** | **N (%)** |
| Croatia | 277 (38.58) | 375 (52.23) | 49 (6.82) | 17 (2.37) |
| Greece | 23 (20.18) | 31 (27.19) | 26 (22.81) | 34 (29.82) |
| Federation of B&H | 27 (16.46) | 60 (36.59) | 47 (28.66) | 30 (18.29) |
| Montenegro | 5 (14.71) | 6 (17.65) | 16 (47.06) | 7 (20.39) |
| North Macedonia | 14 (14.74) | 40 (42.11) | 30 (31.58) | 11 (11.58) |
| Republic of Srpska | 55 (7.18) | 579 (75.59) | 89 (11.62) | 43 (5.61) |
| Serbia | 120 (24.44) | 188 (38.29) | 141 (28.72) | 42 (8.55) |
